# Selenium halide-induced bridge formation in [2.2]paracyclophanes

**DOI:** 10.3762/bjoc.10.266

**Published:** 2014-10-31

**Authors:** Laura G Sarbu, Henning Hopf, Peter G Jones, Lucian M Birsa

**Affiliations:** 1 Department of Chemistry,“Al. I. Cuza” University of Iasi, 11 Carol I Bv., RO-700506 Iasi, Romania; 2 Institute of Organic Chemistry, Technical University of Braunschweig, Hagenring 30, D-38106 Braunschweig, Germany; 3 Institute of Inorganic and Analytical Chemistry, Technical University of Braunschweig, Hagenring 30, D-38106 Braunschweig, Germany

**Keywords:** acetylenes, dienes, [2.2]paracyclophane, selenium halides

## Abstract

An addition/elimination sequence of selenium halides to *pseudo-geminally* bis(acetylene) substituted [2.2]paracyclophanes leads to new bridges with an *endo*-*exo*-diene substructure. The reactions have been found to be sensitive to the substitution of the ethynyl group. The formation of dienes with a zig-zag configuration is related to that observed for non-conjugated cyclic diynes of medium ring size.

## Introduction

Starting with their discovery in 1949, the [2.2]paracyclophane molecule and its derivatives have been intensely studied [1–3]. Of particular interest are the geometry and transannular interactions of these molecules, the study of electrophilic aromatic substitution reactions involving these systems and their ability to form charge-transfer complexes [4–7]. Much attention is also being paid to the development of new functionalized [2.2]paracyclophanes that can be used in asymmetric synthesis [8], while the formation of new bridges, particularly functionalized ones, has been somewhat neglected so far. Functional groups in *pseudo-geminally* substituted [2.2]paracyclophanes often undergo highly specific reactions. This is due to the rigid framework and the short distance between the two aromatic rings within the [2.2]paracyclophane unit. Thus, unsaturated cyclophane bis(esters) undergo intramolecular photocyclization, yielding the corresponding ladderane isomers [9–11]. The ethynyl group is well known for its ability to undergo coupling reactions, making the *pseudo-geminal* bis(acetylene) **1** and its derivatives good candidates for building molecular scaffolding [12–13]. The reaction between bis(acetylene) **1** and other acetylene derivatives has been reported to provide new π-bridges in [2.2]paracyclophane [14].

Furthermore, a new way for bridging [2.2]paracyclophanes has been accomplished by the addition of selenium monochloride to *pseudo-geminally* substituted bis(propargylic) alcohols [15]. Organoselenium chemistry has become widely used in organic synthesis, because of the availability of both electrophilic and nucleophilic selenium species [16]. The fundamental aspects of organoselenium chemistry have been comprehensively described in monographs and review articles [17–20]. For electrophilic selenium species, most of the investigations have been carried out in order to study the addition mechanism of selenium halides to alkenes [21–23]. Less work has been reported on the addition of selenyl reagents to alkynes [24–25]. In contrast to the chemistry of sulfur halides, selenium electrophiles undergo smooth 1,2-additions to triple bonds, leading to the formation of functionalized vinyl selenides [26]. It has been reported that the addition of electrophilic selenium reagents to alkynes preferably yields the corresponding *E*-adducts [27].

In continuation of our investigations on the intramolecular interaction of chemically disturbed functional groups in *pseudo-geminally* substituted [2.2]paracyclophanes, we report here the results of the addition reactions of selenium halides to *pseudo-geminal* bis(acetylenes).

## Results and Discussion

**Scheme 1:**
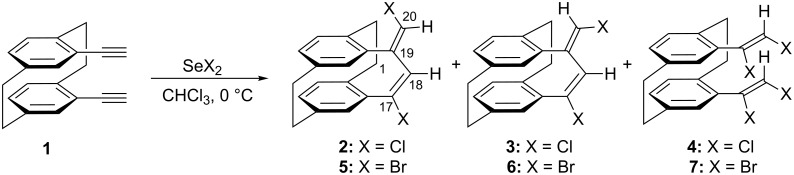
Reactions of selenium dichloride and selenium dibromide with *pseudo-geminal* bis(acetylene) **1**.

Following our interest in the introduction of new bridges to [2.2]paracyclophanes, we decided to investigate a double addition reaction of 1 equiv of selenium dichloride to both triple bonds of 4,13-bis(ethynyl)[2.2]paracyclophane **1**; a bis(vinyl)selenide bridge should result from this interaction. Thus, by reacting **1** with one equivalent of in situ prepared SeCl_2_ [28], in chloroform at 0 °C, a mixture of unexpected addition products has been obtained (Scheme 1). After separation by column chromatography, we assigned the structures of isomeric cyclic dienes **2** and **3** and the tetrachloro derivative **4** on the basis of 2D NMR studies and mass spectrometric analysis. The configuration of compound **2** as a (17*E*,19*E*)-diene was established from the mutual NOEs between H18 and H20. These products were obtained in a 1:1.5:0.5 ratio and an isolated yield of 72% (Table 1, entry 1).

**Table 1:** Addition reactions to bis(acetylene) **1**.

Entry	SeX_2_, equiv	Ratio of products			Yield^a^

		**2**	**3**	**4**	

1	SeCl_2_, 1 equiv	1	1.5	0.5	72
2	SeCl_2_, 2 equiv	1	1.3	–	70

		**5**	**6**	**7**	

3	SeBr_2_, 1 equiv	1	1.4	0.3	76
4	SeBr_2_, 2 equiv	1	1.3	–	73

^a^Total isolated yield.

Analogously, the reaction of **1** with one equivalent of in situ prepared SeBr_2_ [28], in chloroform at 0 °C, provided a mixture of isomeric cyclic dienes **5** and **6**, and the tetrabromo derivative **7** (Scheme 1, Table 1, entry 3) in 76% yield. Again, the structures of these compounds, as well as the configuration of diene **5**, have been established on the basis of 2D NMR studies, mass spectrometry analysis and a mutual NOE between H18 and H20. The relative *Z* stereochemistry of tetrabromo derivative **7** was unambiguously established by X-ray analysis; however, there is a disordered carbon–bromine bond and the structure could not be completely refined. The ratio of **5** and **6** was determined as 1:1.4. It is interesting to note that the synthesis of the [2.3.2](1,2,4)cyclophane derivative **5** has been previously reported as the result of bromine addition to bis(acetylene) **1**, in 87% yield [13].

In both experiments, the formation of elemental red selenium was observed. The unexpected reaction products result from the equimolar interaction of bis(acetylene) **1** with selenium dihalides; the tetrahalide derivatives **4** and **7**, in particular, prompted us to investigate the interaction of **1** with 2 equiv of these selenium derivatives. Under the same experimental conditions, using 2 equiv of selenium dichloride or selenium dibromide, we isolated only the [2.3.2](1,2,4)cyclophane derivatives **2**, **3** and **5**, **6**, respectively; no traces of tetrahalides **4** and **7** were detected (Table 1, entries 2 and 4). Although unexpected, the lack of tetrahalides from these experiments proved later to be of significant importance for the reaction mechanism of selenium dihalide addition to *pseudo-geminal* bis(acetylene) **1**.

**Scheme 2:**
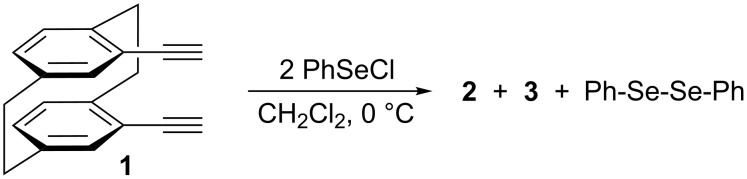
Reaction of phenylselenyl chloride with *pseudo-geminal* bis(acetylene) **1**.

The unexpected formation of [2.3.2](1,2,4)cyclophane derivatives **2**, **3** and **5**, **6**, then induced us to investigate the addition reaction of phenylselenyl chloride to 4,13-bis(ethynyl)[2.2]paracyclophane. Surprisingly, the addition of 2 equiv of PhSeCl to bis(acetylene) **1** again provided a mixture of dienes **2** and **3** along with diphenyl diselenide, in a 70% total isolated yield (Scheme 2).

The ratio of isomeric dienes **2** and **3** was again 1:1.5, as resulted from the SeCl_2_ addition to 4,13-bis(ethynyl)[2.2]paracyclophane. Moreover, an important experimental observation was the fact that diphenyl diselenide was formed in an equimolar amount with the isomeric dienes. This indicates a common intermediate for the reaction products from which elimination of diphenyl diselenide provides dienes **2** and **3**.

**Scheme 3:**
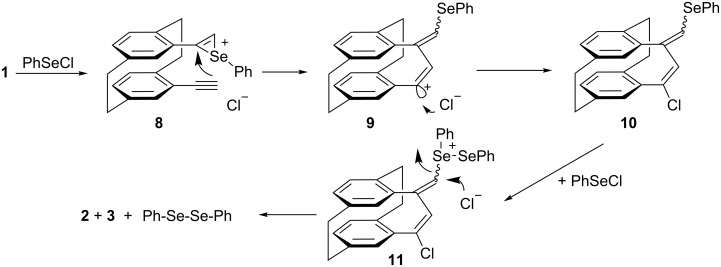
Plausible reaction mechanism for the addition of phenylselenyl chloride to *pseudo-geminal* bis(acetylene) **1**.

Based on previously reported investigations on the mechanism of the reaction of phenylselenyl chloride with selected sterically hindered alkenes [29], the most probable mechanism involves, in a first step, the addition of one equivalent of PhSeCl to one of the triple bonds of **1** resulting in the formation of episelenonium ion **8** (Scheme 3). The episelenonium ion **8** should equilibrate with the ring-opened form, a benzylic type carbocation; the interaction of this intermediate with the opposing ethynyl substituent provides adduct **9**. For steric reasons the chlorine anion attack from "outside" leading to intermediate **10**. The reaction of **10** with the second equivalent of PhSeCl leads to selenonium ion **11**; once the diphenyl diselenide leaving group is formed, the addition of chloride counter-anions from both directions is accompanied by the formation of [2.3.2](1,2,4)cyclophane derivatives **2** and **3**. Most probably compound **3** is formed under kinetic control, diene **2** being thermodynamically more stable.

With regard to the addition of selenium dihalides to bis(acetylene) **1**, the reaction mechanism should follow a similar course, consisting of the formation of a selenonium ion of type **10** rather than the addition of the selenium electrophiles to the second triple bond. This involves elimination of diselenium dihalides with the formation of dienes **2**, **3** and **5**, **6**. The formation of diselenium dihalides appears to be correlated with the presence of tetrahalides **4** and **7** among the reaction products when only one equivalent of selenium dihalides is used. Diselenium dihalides could be the source for the remaining two halogen atoms either by disproportionation, which generates molecular chlorine or bromine [30], or/and by decomposition (e.g., Se_2_Br_2_ + Br^−^ → BrSeSe^−^ + Br_2_). As mentioned before, we always noticed the formation of elemental red selenium. These assumptions are supported by the outcome of the reaction when 2 equiv of selenium dihalides were used (Table 1, entries 2 and 4).

The fact that the ratio of the isomeric dienes is almost identical with those determined for entries 1 and 3, together with the lack of tetrahalide derivatives, suggests that the second equivalent of selenium dihalide acts as described in Scheme 3 for phenylselenyl chloride. This reaction appears to be faster than the involvement of diselenium dihalides in generation of tetrahalides **4** and **7** from incompletely reacted starting materials. Although our synthetic procedure involves an addition/elimination protocol of selenium derivatives, the formation of isomeric *endo*-*exo*-dienes **2**, **3** and **5**, **6** resembles the zig-zag cyclizations of nonconjugated cyclic diynes of medium ring size reported by Gleiter [31–32].

**Scheme 4:**
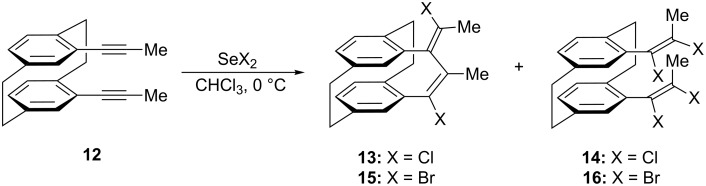
Reactions of selenium dichloride and selenium dibromide with 4,13-bis(propyn-1-yl)[2.2]paracyclophane (**12**).

**Figure 1:**
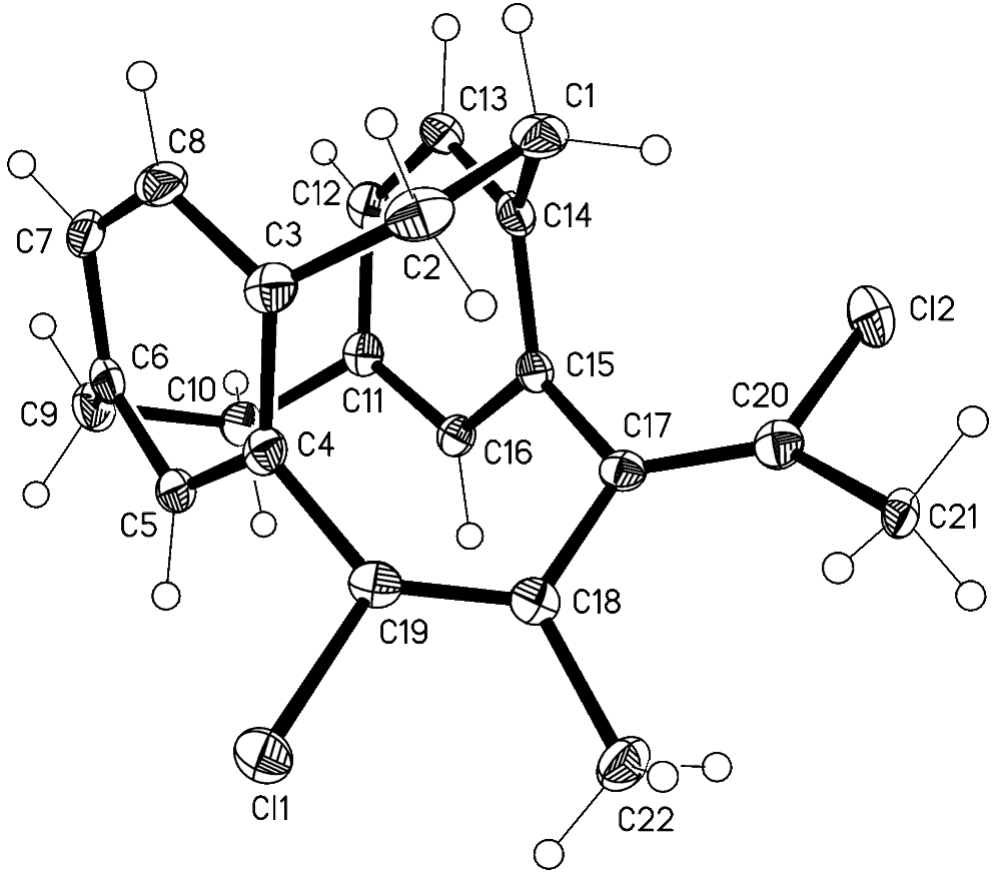
Molecular structure of compound **13**. Ellipsoids represent 50% probability levels. Selected molecular dimensions (Å, °): C17–C20 1.337(3), C18–C19 1.341(3), C18–C19–C4 128.9(2), C17–C20–C21 129.3(2).

In order to check the limits of these selenium-mediated intramolecular interactions, we decided to extend the study to another *pseudo-geminal* bis(acetylene), 4,13-bis(propyn-1-yl)[2.2]paracyclophane **12** (Scheme 4). Thus, by reacting bis(acetylene) (**12**) with 1 equiv of selenium dichloride we isolated only the (17*E*,19*E*)-diene **13** and tetrachloride derivative **14** in 76% yield (Table 2, entry 1). The lack of isomeric diene (17*E*,19*Z*) could be explained as the result of steric hindrance induced by the presence of methyl groups at the acetylenic carbon atoms. This forces the addition of a chloride anion to a methylated intermediate of type **11** to take place in the way that provides only the thermodynamically stable (17*E*,19*E*)-[2.3.2](1,2,4)cyclophane derivative **13**. The structure of this compound was unambiguously proved by X-ray crystallography (Figure 1). A colourless tablet 0.35 × 0.2 × 0.08 mm was used to record intensity data to 2θ 56.6° on an Oxford Diffraction Xcalibur E diffractometer using monochromated Mo *K*α radiation (λ = 0.71073 Å). Crystal data: C_22_H_20_Cl_2_, monoclinic, *P*21/c, *a* = 9.0685(3), *b* = 8.0240(3), *c* = 23.1441(8) Å, β = 93.423(3)° (at 100 K), *Z* = 4. Structure refinement: The structure was treated as a non-merohedral twin (by 180° rotation about the a axis). Refinement on *F*^2^ using the program SHELXL-97 [33] proceeded to *wR*2 0.117 (all data), *R*1 0.046 (*F* > 4σ(*F*)), twinning ratio 0.0481(6), *S* = 1.05, Δρ = 0.4 e Å^–3^. CCDC-997241 contain the supplementary crystallographic data for compound **13**. These data can be obtained free of charge from the Cambridge Crystallographic Data Centre via http://www.ccdc.cam.ac.uk/data_request/cif. Furthermore, the reaction of **12** with 1 equiv of selenium dibromide follows the same course, providing only (17*E*,19*E*)-diene **15** and tetrabromide derivative **16** in 64% yield (Table 2, entry 4).

**Table 2:** Addition reactions to 4,13-bis(propyn-1-yl)[2.2]paracyclophane **12**.

Entry	Selenium electrophile, equiv	Ratio of products		Yield^a^

		**13**	**14**	

1	SeCl_2_, 1 equiv	1	0.6	76
2	SeCl_2_, 2 equiv	1	–	70
3	PhSeCl, 2 equiv	1^b^	–	56

		**15**	**16**	

4	SeBr_2_, 1 equiv	1	0.6	64
5	SeBr_2_, 2 equiv	1	–	62

^a^Total isolated yield. ^b^Along with an equimolar amount of diphenyl diselenide.

In support of the proposed reaction mechanism, the reaction of 4,13-diyne **12** with 2 equivalents of selenium dichloride or selenium dibromide provided only (17*E*,19*E*)-diene **13** and **15**; again, no traces of tetrahalide derivatives were identified (Table 2, entries 2 and 5). The addition of phenylselenyl chloride to **12** provides additional support to the proposed reaction mechanism, as only diene **13**, along an equimolar amount of diphenyl diselenide, was obtained (Table 2, entry 3).

## Conclusion

We present here an addition/elimination sequence of selenium halides to *pseudo-geminally* bis(acetylene) substituted [2.2]paracyclophanes that leads to a new bridge with an *endo*-*exo*-diene substructure. The reactions have been found to be sensitive to the substitution of the acetylenic bond.

## Supporting Information

File 1 Detailed experimental procedures, supplementary spectroscopic and X-ray data.
