# Supplementary material for: Selenium halide-induced bridge formation in [2.2]paracyclophanes
Source: Beilstein J Org Chem. 2014 Oct 31;10:2550–5. doi: 10.3762/bjoc.10.266 (PMC4222412; doi:10.3762/bjoc.10.266)
Supplement: File 1 — Detailed experimental procedures, supplementary spectroscopic and X-ray data. [file Beilstein_J_Org_Chem-10-2550-s001.pdf]

Supporting Information

for

Selenium halide-induced bridge formation in [2.2]paracyclophanes

Laura G. Sarbu[1], Henning Hopf [2], Peter G. Jones [3], and Lucian M. Birsa*[1,2]

Address: [1]Department of Chemistry,“Al. I. Cuza” University of Iasi, 11 Carol I Bv., RO-700506 Iasi, Romania, [2]Institute of Organic Chemistry, Technical University of Braunschweig, Hagenring 30, D-38106 Braunschweig, Germany and [3]Institute of Inorganic and Analytical Chemistry, Technical University of Braunschweig, Hagenring 30, D-38106 Braunschweig

Email: Lucian M. Birsa* - lbirsa@uaic.ro

* Corresponding author

Experimental and X-ray spectral data

General Remarks: Melting points: Büchi 510, uncorrected. IR: Bruker Tensor 27 with ATR. ^{1}H and ^{13}C NMR: Bruker spectrometers at 300, 400, or 500 MHz in $CDCl_3$ with TMS as internal standard at room temp. Chemical shifts are reported in ppm downfield from tetramethylsilane. MS: Finnigan MAT 90X, electron impact

(EI). Elemental analyses: CE440 Elemental Analyser; the results were found to be in good agreement (±0.32%) with the calculated values. All reagents were commercially available and used without further purification. 4,13-Bis(ethynyl)[2.2]paracyclophane **1** [1] and 4,13-bis(propyn-1-yl)[2.2]paracyclophane **12** [2] were synthesized according to the literature procedures.

Reaction of selenium dichloride with *pseudo-geminal* bis(acetylene) 1

Protocol A: To a solution of *pseudo-geminal* bis(acetylene) **1** (256 mg, 1 mmol) in $CHCl_3$ (70 mL) a freshly prepared solution of $SeCl_2$ (150 mg, 1 mmol) in $CHCl_3$ (20 mL) [3] was added dropwise at 0 °C. The reaction mixture was allowed to warm to room temperature and then stirred at this temperature for 6 h. The solvent was removed under vacuum and the residue purified by PTLC using CH_2Cl_2/pentane (2:5) as eluent to provide compounds **2**, **3** and **4**.

Compound 2: White solid; mp 110-112 °C; R_f 0.56; yield 24% (77 mg); IR 3060, 3008, 2918, 2850, 1616, 1577, 1453, 1334, 868, 811, 776, 634, 541 cm^{-1}; ^{1}H NMR ($CDCl_3$, 400 MHz) δ 2.79-2.92 (m, 2 H, CH_2), 2.98-3.16 (m, 5 H, CH_2), 3.27-3.34 (m, 1 H, CH_2), 6.26 (d, J = 1.7 Hz, 1 H, CH_{ar}), 6.33-6.34 (m, 1 H, CH_{ar}), 6.43 (s, 1 H, CH), 6.46 (d, J = 1.7 Hz, 1 H, CH_{ar}), 6.47-6.48 (m, 1 H, CH_{ar}), 6.50-6.52 (m, 2 H, CH_{ar}), 6.76 (s, 1 H, CH) ppm; ^{13}C NMR ($CDCl_3$, 100 MHz) δ 32.5

1. Bondarenko, L.; Dix, I.; Hinrich, H.; Hopf, H. *Synthesis,* **2004,** 2751–2759.
2. Bondarenko, L.; Hentschel, S.; Greiving, H.; Grunenberg, J.; Hopf, H.; Dix, I.; Jones, P. G.; Ernst, L. *Chem. Eur. J.*, **2007,** *13*, 3950–3963.
3. Amosova, S. V.; Martynov, A. V.; Mahaeva, N. A.; Belozerova, O. V.; Penzik, M. V.; Albanov, A. I.; Yarosh, O. G.; Voronkov, M. G. *J. Organomet. Chem*. **2007**, *692*, 946-952.

(CH_2), 33.3 (CH_2), 34.8 (CH_2), 35.2 (CH_2), 120.6 (CH), 127.5 (CH), 133.1 (CH), 133.4 (CH), 133.9 (C), 134.2 (CH), 134.3 (CH), 134.5 (CH), 135.2 (CH), 135.6 (CH), 137.2 (C), 137.8 (C), 138.4 (C), 140.3 (C), 140.4 (C), 141.1 (C) ppm; MS (EI) *m/z* (%) 328 (46) [M^++2] for ^{37}Cl, 326 (74) [M^+] for ^{35}Cl, 291 (50), 255 (100), 239 (58), 226 (21), 175 (26), 163 (27), 128 (31).

Compound 3: White solid; mp 138-139 °C; R_f 0.72; yield 36% (117 mg); IR 3078, 3072, 2930, 2855, 1614, 1563, 1453, 1341, 1203, 901, 876, 833, 789, 716, 623, 545 cm^{-1}; ^{1}H NMR ($CDCl_3$, 400 MHz) δ 2.77-2.90 (m, 2 H, CH_2), 3.00-3.19 (m, 5 H, CH_2), 3.29-3.36 (m, 1 H, CH_2), 5.85 (d, *J* = 0.8 Hz, 1 H, CH), 6.25-6.30 (m, 1 H, CH_{ar}), 6.30-6.34 (m, 1 H, CH_{ar}), 6.41-6.43 (m, 2 H, CH_{ar}), 6.49-6.52 (m, 2 H, CH_{ar}), 7.33 (d, *J* = 0.8 Hz, 1 H, CH) ppm; ^{13}C NMR ($CDCl_3$, 100 MHz) δ 32.4 (CH_2), 33.4 (CH_2); 34.5 (CH_2), 35.1 (CH_2), 117.1 (CH), 124.1 (CH), 133.1 (CH), 133.4 (CH), 134.1 (CH), 134.3 (CH), 135.3 (CH), 135.9 (C), 136.5 (CH), 137.3 (C), 137.4 (C), 137.7 (C), 138.6 (C), 139.5 (C), 140.2 (C), 140.5 (C) ppm; MS (EI) *m/z* (%) 328 (47) [M^++2] for ^{37}Cl, 326 (71) [M^+] for ^{35}Cl, 291 (68), 255 (100), 239 (67), 226 (24), 175 (13), 163 (27), 128 (34).

Compound 4: Yellow solid; mp 128-129 °C; R_f 0.47; yield 12% (47 mg); IR 2923, 2852, 1585, 1466, 1233, 946, 907, 848, 793, 729, 647, 542 cm^{-1};^{1}H NMR ($CDCl_3$, 500 MHz) δ 2.99-3.05 (m, 2 H, CH_2), 3.06-3.15 (m, 4 H, CH_2), 3.55-3.62 (m, 2 H, CH_2), 6.50 (s, 2 H, CH), 6.65-6.73 (m, 6 H, CH_{ar}) ppm; ^{13}C NMR ($CDCl_3$, 125 MHz) δ 34.7 (CH_2), 35.0 (CH_2), 119.4 (CH), 129.5 (CH), 133.7 (CH), 134.3 (C), 134.9 (C), 136.0 (CH), 137.2 (C), 139.2 (C) ppm; MS (EI) *m/z* (%) 400

(41) [M^++2] for ^{37}Cl, 398 (100) [M^+] for ^{35}Cl, 362 (18), 325 (21), 289 (16), 224 (24), 183 (32), 163 (60).

Protocol B: This follows the same experimental procedure as described under ***Protocol A*** using a solution containing 2 eq. of $SeCl_2$ (300 mg, 2 mmol). Purification by PTLC provided only compounds **2** (98 mg, 30%) and **3** (130 mg, 40%).

Reaction of selenium dibromide with *pseudo-geminal* bis(acetylene) 1

Protocol A: To a solution of *pseudo-geminal* bis(acetylene) **1** (256 mg, 1 mmol) in $CHCl_3$ (50 mL) a freshly prepared solution of $SeBr_2$ (239 mg, 1 mmol) in $CHCl_3$ (15 mL) [3] was added dropwise at 0 °C. The reaction mixture was allowed to warm to room temperature and then stirred at this temperature for 6 h. The solvent was removed under vacuum and the residue purified by PTLC using CH_2Cl_2/pentane (1:5) as eluent to provide compounds **5**, **6** and **7**.

Compound 5: Yellow solid; mp 138-139 °C; R_f 0.49; yield 28% (117 mg); IR 2958, 2929, 2891, 2851, 1586, 1547, 1479, 1332, 1236, 875, 773, 750, 617, 590 cm^{-1}; ^{1}H NMR ($CDCl_3$, 400 MHz) δ 2.79-2.91 (m, 2 H, CH_2), 2.97-3.14 (m, 5 H, CH_2), 3.26-3.34 (m, 1 H, CH_2), 6.24 (d, J = 1.7 Hz, 1 H, CH_{ar}), 6.30-6.31 (m, 1 H, CH_{ar}), 6.45 (d, J = 1.7 Hz, 1 H, CH_{ar}), 6.45-6.47 (m, 1 H, CH_{ar}), 6.47-6.49 (m, 2 H, CH_{ar}), 6.62 (s, 1 H, CH), 7.00 (s, 1 H, CH) ppm; ^{13}C NMR ($CDCl_3$, 100 MHz) δ 32.4 (CH_2), 33.2 (CH_2), 34.7 (CH_2), 35.1 (CH_2), 111.4 (CH), 124.3 (C), 131.9

(CH), 133.1 (CH), 133.4 (CH), 134.2 (CH), 134.5 (CH), 135.0 (CH), 135.2 (CH), 135.8 (C), 137.3 (C), 138.2 (C), 138.8 (C), 140.3 (C), 140.4 (C), 144.4 (C) ppm; MS (EI) *m/z* (%) 418 (22) $[M^{+}+2]$ for ^{81}Br, 416 (39) $[M^{+}]$ for ^{79}Br, 335 (24), 255 (100), 239 (41), 226 (14), 128 (42).

Compound 6: White solid; mp 121-122 °C; R_f 0.57; yield 40% (167 mg); IR 3078, 3041, 2925, 2892, 2852, 1606, 1559, 1482, 1304, 1230, 814, 750, 702, 663, 618, 598, 574 cm^{-1}; ^{1}H NMR ($CDCl_3$, 400 MHz) δ 2.76-2.89 (m, 2 H, CH_2), 2.98-3.17 (m, 5 H, CH_2), 3.26-3.37 (m, 1 H, CH_2), 5.89 (d, J = 0.8 Hz, 1 H, CH), 6.25-6.27 (m, 1 H, CH_{ar}), 6.27-6.29 (m, 1 H, CH_{ar}), 6.39-6.42 (m, 2 H, CH_{ar}), 6.46-6.49 (m, 2 H, CH_{ar}), 7.51 (d, J = 0.8 Hz, 1 H, CH) ppm; ^{13}C NMR ($CDCl_3$, 100 MHz) δ 32.6 (CH_2), 33.7 (CH_2), 34.8 (CH_2), 35.3 (CH_2), 107.5 (CH), 129.3 (C), 130.1 (CH), 133.4 (CH), 133.6 (CH), 134.4 (CH), 134.7 (CH), 135.2 (CH), 136.5 (CH), 137.0 (C), 137.2 (C), 139.2 (C), 139.4 (C), 140.5 (C), 140.7 (C), 142.1 (C) ppm; MS (EI) *m/z* (%) 418 (19) $[M^{+}+2]$ for ^{81}Br, 416 (37) $[M^{+}]$ for ^{79}Br, 335 (26), 255 (100), 239 (48), 226 (18), 128 (46).

Compound 7: White solid; mp 166-167 °C; R_f 0.42; yield 8% (46 mg); IR 3073, 2930, 1582, 1431, 1157, 899, 794, 777, 723, 695, 657, 550 cm^{-1}; ^{1}H NMR ($CDCl_3$, 400 MHz) δ 2.94-3.00 (m, 2 H, CH_2), 3.03-3.13 (m, 4 H, CH_2), 3.46-3.53 (m, 2 H, CH_2), 6.64-6.67 (m, 6 H, CH_{ar}), 6.96 (s, 2 H, CH) ppm; ^{13}C NMR ($CDCl_3$, 100 MHz) δ 34.6 (CH_2), 34.9 (CH_2), 112.5 (CH), 129.6 (C), 130.7 (CH), 133.8 (CH), 135.9 (CH), 137.0 (C), 137.3 (C), 139.3 (C) ppm; MS (EI) *m/z* (%) 577 (9) $[M^{+}+2]$ for ^{81}Br, 575 (11) $[M^{+}]$ for ^{79}Br, 495 (18), 416 (63), 337 (39), 286 (25), 256 (48), 241 (100), 226 (32).

Protocol B: This follows the same experimental procedure as described under ***Protocol A*** using a solution containing 2 eq. of $SeBr_2$ (478 mg, 2 mmol). Purification by PTLC provided only compounds **5** (133 mg, 32%) and **6** (171 mg, 41%).

Reaction of phenylselenyl chloride with *pseudo-geminal* bis(acetylene) 1

To a solution of *pseudo-geminal* bis(acetylene) **1** (256 mg, 1 mmol) in CH_2Cl_2 (10 mL) a solution of PhSeCl (383 mg, 2 mmol) in CH_2Cl_2 (10 mL) was added dropwise at 0 °C. The reaction mixture was allowed to warm to room temperature and then stirred at this temperature for 6 h. The solvent was removed under vacuum and the residue purified by PTLC using CH_2Cl_2/pentane (3:5) as eluent to provide diphenyl diselenide (109 mg, 35%) along with the compounds **2** (46 mg, 14%) and **3** (68 mg, 21%).

Reaction of selenium dichloride with 4,15-bis(propyn-1-yl)[2.2]paracyclophane 12

Protocol A: To a solution of bis(acetylene) **12** (284 mg, 1 mmol) in $CHCl_3$ (70 mL) a freshly prepared solution of $SeCl_2$ (150 mg, 1 mmol) in $CHCl_3$ (20 mL) was added dropwise at 0 °C. The reaction mixture was allowed to warm to room temperature and then stirred at this temperature for 6 h. The solvent was

removed under vacuum and the residue purified by PTLC using CH_2Cl_2/pentane (1:4) as eluent to provide compounds **13** and **14**.

Compound 13: White solid; mp 177-178 °C; R_f 0.42; yield 47% (166 mg). IR 2923, 2852, 1576, 1554, 1433, 1083, 940, 793, 728, 622, 598, 549 cm^{-1}; ^1H NMR ($CDCl_3$, 300 MHz) δ 2.43 (s, 3 H, CH_3), 2.47 (s, 3 H, CH_3), 2.70-3.20 (m, 8 H, CH_2), 6.28-6.31 (m, 1 H, CH_{ar}), 6.38-6.42 (m, 2 H, CH_{ar}), 6.42-6.49 (m, 3 H, CH_{ar}) ppm; ^{13}C NMR ($CDCl_3$, 75 MHz) δ 23.9 (CH_3), 27.2 (CH_3), 32.5 (CH_2), 34.6 (CH_2), 35.4 (CH_2), 35.6 (CH_2), 130.4 (C), 132.2 (CH), 132.7 (CH), 133.4 (CH), 134.0 (C), 134.5 (CH), 135.1 (C), 135.6 (CH), 137.6 (CH), 137.7 (C), 138.1 (C), 138.3 (C), 139.6 (C), 139.7 (C), 140.4 (C), 140.7 (C) ppm; MS (EI) *m/z* (%)356 (62) [M^++2] for ^{37}Cl, 354 (100) [M^+] for ^{35}Cl, 319 (60), 283 (69), 269 (44), 253 (45), 239 (34), 177 (88), 141 (33) 126 (37).

Compound 14: White solid; mp 144-145 °C; R_f 0.47; yield 29% (100 mg). IR 2923, 2849, 1583, 1431, 1087, 895, 803, 784, 753, 715, 637, 586 cm^{-1}; ^1H NMR ($CDCl_3$, 400 MHz) δ 2.35 (s, 6 H, $2CH_3$), 2.89-3.12 (m, 6 H, CH_2), 3.41 (m, 2 H, CH_2), 6.62-6.73 (m, 6 H, CH_{ar}) ppm; ^{13}C NMR ($CDCl_3$, 100 MHz) δ 24.9 (CH_3), 35.2 (CH_2), 36.0 (CH_2), 126.6 (C), 131.7 (CH), 133.9 (CH), 134.8 (CH), 134.9 (C), 136.4 (C), 138.2 (C), 139.3 (C) ppm; MS (EI) *m/z* (%) 428 (49) [M^++2] for ^{37}Cl, 426 (100) [M^+] for ^{35}Cl, 390 (15), 353 (17), 317 (15), 252 (18), 211 (29), 191 (59).

Protocol B: This follows the same experimental procedure as described under ***Protocol A*** using a solution containing 2 eq. of $SeCl_2$ (300 mg, 2 mmol). Purification by column chromatography provided only compound **13** (306 mg, 72%).

Reactions of selenium dibromide with 4,15-bis(propyn-1-yl)[2.2]paracyclophane 12

Protocol A: To a solution of bis(acetylene) **12** (284 mg, 1 mmol) in $CHCl_3$ (50 mL) a freshly prepared solution of $SeBr_2$ (239 mg, 1 mmol) in $CHCl_3$ (15 mL) was added dropwise at 0 °C. The reaction mixture was allowed to warm to room temperature and then stirred at this temperature for 6 h. The solvent was removed under vacuum and the residue purified by PTLC using CH_2Cl_2/pentane (1:5) as eluent to provide compounds **15** and **16**.

Compound 15: White solid; mp 143-144 °C; R_f 0.37; yield 40% (178 mg). IR 2988, 2921, 2849, 1570, 1482, 1431, 1371, 1122, 1033, 933, 810, 786, 715, 643, 611, 570 cm^{-1}; ^1H NMR ($CDCl_3$, 400 MHz) δ 2.16 (s, 3 H, CH_3), 2.63 (s, 3 H, CH_3), 2.66-2.76 (m, 1 H, CH_2), 2.78-2.91 (m, 2 H, CH_2), 2.92-3.23 (m, 5 H, CH_2), 6.27-6.31 (m, 1 H, CH_{ar}), 6.35-6.39 (m, 1 H, CH_{ar}), 6.39-6.45 (m, 4 H, CH_{ar}) ppm; ^{13}C NMR ($CDCl_3$, 100 MHz) δ 27.8 (CH_3), 29.4 (CH_3), 32.4 (CH_2), 34.9 (CH_2), 35.3 (CH_2), 35.6 (CH_2), 123.5 (C), 129.3 (C), 132.3 (CH), 132.6 (CH), 133.3 (CH), 134.7 (CH), 135.6 (CH), 137.1 (C), 137.4 (C), 137.6 (CH), 139.8 (C), 140.1

(C), 140.2 (C), 140.3 (C), 142.8 (C) ppm; MS (EI) *m/z* (%) 446 (31) [M^++2] for ^{81}Br, 444 (66) [M^+] for ^{79}Br, 365 (37), 283 (97), 269 (100), 253 (45), 239 (35), 142 (50), 126 (31).

Compound 16: White solid; mp 202-203 °C; R_f 0.35; yield 24% (145 mg). IR 3030, 2998, 2949, 2925, 2852, 1579, 1476, 1431, 1370, 1086, 893, 818, 782, 724, 617, 584 cm^{-1}; ^{1}H NMR ($CDCl_3$, 400 MHz) δ 2.55 (s, 6 H, CH_3), 2.92-3.03 (m, 4 H, CH_2), 3.05-3.12 (m, 2 H, CH_2), 3.55-3.62 (m, 2 H, CH_2), 6.62-6.66 (m, 2 H, CH_{ar}), 6.69 (dd, J = 7.6, 1.7 Hz, 2 H, CH_{ar}), 6.79 (d, J = 1.7 Hz, 2 H, CH_{ar}) ppm; ^{13}C NMR ($CDCl_3$, 100 MHz) δ 30.8 (CH_3), 35.2 (CH_2), 35.8 (CH_2), 117.9 (C), 118.2 (C), 133.0 (CH), 134.0 (CH), 134.6 (CH), 135.1 (C), 138.4 (C), 138.9 (C) ppm; MS (EI) *m/z* (%) 605 (5) [M^++2] for ^{81}Br, 603 (10) [M^+] for ^{79}Br, 523 (14), 444 (67), 365 (45), 314 (20), 284 (63), 269 (100), 254 (30).

Protocol B: This follows the same experimental procedure as described under ***Protocol A*** using a solution containing 2 eq. of $SeBr_2$ (478 mg, 2 mmol). Purification by column chromatography provided only compound **15** (276 mg, 62%).

Reaction of phenylselenyl chloride with 4,15-bis(propyn-1-yl)[2.2]paracyclophane 12

To a solution of bis(acetylene) **12** (284 mg, 1 mmol) in CH_2Cl_2 (20 mL) a solution of PhSeCl (383 mg, 2 mmol) in CH_2Cl_2 (10 mL) was added dropwise at

0 °C. The reaction mixture was allowed to warm to room temperature and then stirred at this temperature for 6 h. The solvent was removed under vacuum and the residue purified by PTLC using CH_2Cl_2/pentane (1:10) as eluent to provide diphenyl diselenide (87 mg, 28%) along with an equimolar amount of **13** (92 mg, 28%).
